# Efficacy of Motivational Interviewing and Brief Interventions on tobacco use among healthy adults: A systematic review of randomized controlled trials*

**DOI:** 10.17533/udea.iee.v40n3e03

**Published:** 2023-02-08

**Authors:** Rajesh Kumar, Maya Sahu, Tamar Rodney

**Affiliations:** 1 Associate Professor, College of Nursing, All India Institute of Medical Sciences (AIIMS) Rishikesh Uttarakhand, India. Email: rajeshrak61@gmail.com. Corresponding author All India Institute Of Medical Sciences College of Nursing All India Institute of Medical Sciences Rishikesh Uttarakhand India rajeshrak61@gmail.com; 2 Assistant Professor. College of Nursing Teerthanker Mahaveer University, Moradabad, Uttar Pradesh, India. Email: mayamonsahu@gmail.com College of Nursing Teerthanker Mahaveer University Moradabad Uttar Pradesh India mayamonsahu@gmail.com; 3 Assistant Professor. Johns Hopkins School of Nursing, Baltimore MD, USA. Email: trodney1@jhu.edu Johns Hopkins School of Nursing Baltimore MD USA trodney1@jhu.edu

**Keywords:** motivational interviewing, tobacco use cessation, tobacco use, adult., entrevista motivacional, cese del uso de tabaco, uso de tabaco, adulto, entrevista motivacional, abandono do uso de tabaco, uso de tabaco, adulto.

## Abstract

**Objective.:**

To assess the effectiveness of a brief intervention and motivational interviewing in reducing the use of different tobacco-related products in adults

**Methods.:**

For this systematic review, PubMed, Web of Science, and PsychINFO databases were electronically searched for randomized controlled trials on the effect of a brief intervention and / or motivational interview on tobacco reduction among healthy adults published between January 1, 2011 to January 1, 2021. Data from eligible studies were extracted and analyzed. CONSORT guidelines were used to assess the quality of the studies by two reviewers for the included studies. The titles and abstracts of the search results were screened and reviewed by two independent reviewers for eligibility criteria per the inclusion and exclusion criteria. Cochrane review criteria were used to assess the risk of bias in included studies.

**Results.:**

A total of 12 studies were included in the final data extraction of 1406 studies. The brief intervention and motivational interviewing showed varied effects on tobacco use reduction among adults at different follow-ups. Seven of the 12 studies (58.3%) reported a beneficial impact on reducing tobacco use. Pieces of evidence on biochemical estimation on tobacco reduction are limited compared to self-reports, and varied results on quitting and tobacco cessation with different follow-ups.

**Conclusion.:**

The current evidence supports the effectiveness of a brief intervention and motivational interviewing to quit tobacco use. Still, it suggests using more biochemical markers as outcome measures to reach an intervention-specific decision**.** While more initiatives to train nurses in providing non-pharmacological nursing interventions, including brief interventions, are recommended to help people quit smoking.

## Resumen

**Objetivo.:**

Evaluar la eficacia de una intervención breve y de la entrevista motivacional para reducir el consumo de diferentes productos relacionados con el tabaco en adultos.

**Métodos.:**

Para esta revisión sistemática, se buscaron en las bases de datos PubMed, Web of Science y PsychINFO ensayos controlados aleatorizados sobre el efecto de una intervención breve y/o una entrevista motivacional en la reducción del consumo de tabaco entre adultos sanos, que hubieran sido publicados entre el 1 de enero de 2011 y el 1 de enero de 2021. Los títulos y los resúmenes de los artículos incluidos fueron evaluados por dos revisores independientes para determinar los criterios de elegibilidad, se analizó la calidad de los estudios con la guía CONSORT y se utilizaron los criterios de Cochrane para evaluar el riesgo de sesgo.

**Resultados.:**

Se incluyeron un total de 12 de los 1406 estudios que arrojó la búsqueda. La intervención breve y la entrevista motivacional mostraron efectos variados en la reducción del consumo de tabaco entre los adultos en diferentes seguimientos. Siete de los 12 estudios (58.3%) informaron de un impacto beneficioso en la reducción del consumo de tabaco. La utilización de indicadores bioquímicos de la reducción del consumo de tabaco fueron limitados en comparación con los autoinformes. Los resultados sobre el abandono y la cesación del tabaco fueron variados con diferentes seguimientos**.**

**Conclusión.:**

La evidencia apoyó la efectividad de una intervención breve y de la entrevista motivacional para la cesación del consumo de tabaco. Sin embargo, se sugiere realizar más estudios con marcadores bioquímicos como medidas de resultado para llegar a una decisión específica de la intervención. Se recomienda formar a los enfermeros en la realización de intervenciones de enfermería no farmacológicas, incluidas las intervenciones breves, para ayudar a las personas a dejar de fumar.

## Resumo

**Objetivo:**

. Avaliar a eficácia de uma intervenção breve e entrevista motivacional na redução do uso de diferentes produtos relacionados ao tabaco em adultos.

**Métodos:**

. Para esta revisão sistemática, se buscou nas bases de PubMed, Web of Science e PsychINFO ensaios controlados aleatórios sobre o efeito de uma breve intervenção e/ou entrevista motivacional na redução do uso de tabaco entre adultos saudáveis, publicados entre 1º de janeiro de 2011 e 1º de janeiro de 2021. Os títulos e resumos dos artigos incluídos foram avaliados por dois revisores independentes para critérios de elegibilidade, a qualidade do estudo foi avaliada usando a diretriz CONSORT e os critérios Cochrane foram usados para avaliar o risco de viés.

**Resultados:**

. Um total de 12 dos 1.406 estudos retornados pela busca foram incluídos. Intervenção breve e entrevista motivacional mostraram efeitos mistos na redução do uso de tabaco entre adultos em diferentes acompanhamentos. Sete dos 12 estudos (58.3%) relataram um impacto benéfico na redução do uso de tabaco. O uso de indicadores bioquímicos de redução do uso de tabaco foi limitado em relação ao autorrelato. Os resultados sobre parar de fumar e parar de fumar foram variados com diferentes seguimentos.

**Conclusão:**

. As evidências apoiaram a eficácia de uma intervenção breve e entrevista motivacional para a cessação do uso do tabaco. No entanto, mais estudos com marcadores bioquímicos como medidas de resultados são sugeridos para chegar a uma decisão de intervenção específica. Recomenda-se que os enfermeiros sejam treinados na execução de intervenções de enfermagem não farmacológicas, incluindo intervenções breves, para ajudar as pessoas a parar de fumar.

## Introduction

Tobacco in any form is harmful and affects millions of lives every year.(1) In 2017, 8 million lives were lost due to smoking-related diseases.(2) Tobacco-related deaths are rising even after a decline in tobacco use trends because of the chronic nature of conditions.(3) In 2000, around 33.3% of the global population over 15 years old were current tobacco users.(3) The negative consequences of tobacco use are well known and extend beyond individuals and countries regarding increasing health care expenditure and loss of productive life.(4) The tobacco consumption trend was three times higher in males than females in 2000, which was increased to four times in 2015 and is projected to be five times by 2025.(1,3) Notably, the detrimental effects of tobacco use gravely affected lower socio-economic populations with higher smoking prevalence.(5) However, tobacco use practices are varied and influenced by the locally available tobacco products in the different regions worldwide.(6)

Smoking is one of the modifiable risk factors for many life-threatening health problems, including respiratory and cardiovascular health and genitourinary problems.(7) It has been estimated that 50% of smokers who start smoking in adolescence die due to tobacco-related health problems.(8) Thus, an effective measure to control tobacco addiction is paramount. Implementing a wide range of interventions and strengthening tobacco control policy, including taxation, ban on tobacco use in public places, restriction on advertising of tobacco products, and creating smoke-free zones in educational institutions, brought a substantial decline in tobacco use in recent decades.(4) In addition to government initiatives to curb tobacco use, many pharmacological and non-pharmacological approaches are also involved in reducing tobacco-associated mortality and the burden of diseases.(6,9) Earlier studies reported that using a combination of pharmacologic and non-pharmacologic intervention is highly effective in reducing tobacco use. (10-12) However, non-pharmacological interventions have advantages over pharmacological interventions, including no side effects, long-term behavior changes,(13) knowing the real health hazards of long-term tobacco use, and cost-effective to show higher compliance.(11,12,14)

Non-pharmacologic interventions for tobacco cessation include telephone counseling, individual and group counseling, health care provider interventions, exercise programs, and self-help programs.(12) Brief intervention or motivational interview is a brief yet realistic strategy offered to those who have a low motivation to quit.(15) Brief intervention is goal-directed but non-directive communication designed to improve motivation for change in quit behavior by eliciting feedback to plan for change.(12,16-20) The terms brief intervention (BI) and motivational interview (MI) are used with a common principle of active engagement of the client in the process of reduced use and teaching alternative coping skills.(21) These interventions are based on the philosophy that the client holds a key role in showing commitment and successful recovery.(22) Brief intervention sometimes follows the principles of the motivational interview to motivate the specific behavior of an individual to reduce or quit substance use.(23)

However, these interventions are substantially modified in the delivery approach, format, and content in earlier published work.(12) Brief intervention primarily focuses on present concerns and stressors rather than exploring the historical antecedents of an individual and is conducted by a trained therapist.(20,24) Earlier work on the efficacy of brief intervention reported evidence that brief intervention increases the motivation to quit short-term use.(18,25) However, the evidence on long-term effects of brief interventions is equivocal, with no reduction of tobacco use at three months while higher self-reported abstinence at 1-year post-brief intervention.(26) Conversely, the brief intervention was found to be effective in improving quit rates, prolonging abstinence, and improving self-reported continuous abstinence among smokers at six months(27) and 1-year post-intervention(28) in other work. Still, there is a lack of consistent evidence on brief interventions to reduce use or quit tobacco use among the adult population.

Nurses are an essential attribute of the health care system and play a vital role in delivering various interventions. It is natural to expect that nurses with adequate knowledge and skills in the brief intervention will do more to help their patients quit smoking. This meta-analysis will highlight the need for encouragement and opportunities to nurses to receive training on smoking cessation interventions. In addition, this will be insightful for the nurses to understand the significance of a non-pharmacological intervention to quit smoking. Towards this end, training nurses in the brief intervention using motivational interviews may be helpful to smokers and their families. Consequently, this systematic review aims to assess the effectiveness of the brief intervention in reducing tobacco use among adults. 

## Methods

A literature review was conducted with online databases PubMed, Web of Science, and PsychINFO. A literature search was completed using Boolean operators and truncations for the following key terms: (1) "Brief Intervention, (2) OR Screening and Brief Intervention” "tobacco products” AND (3) “Tobacco OR "tobacco products,” (MESH terms are also included in the search). The problem/disease was tobacco use among adults in the experimental group. The primary outcomes of interest were cessation in tobacco use, motivation/readiness to quit, reduction in tobacco quantity, days, abstinence days, quit attempts, and point prevalence measured by self-reported methods or biochemical verification at different intervals.

**Figure 1:**
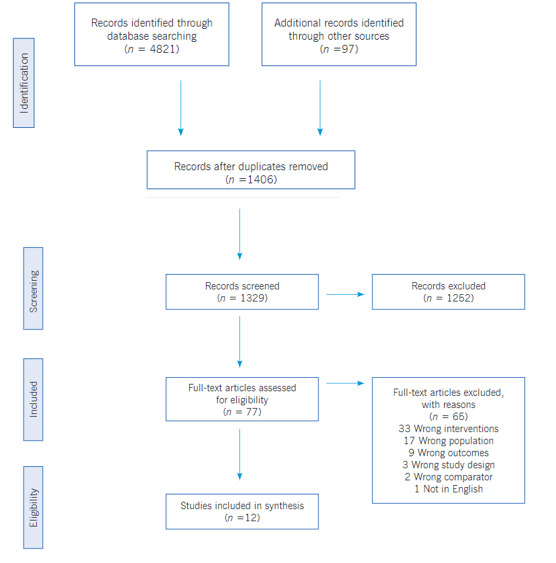
PRISMA Flow Diagram

Selection criteria and data extraction. The inclusion criteria for the studies included in this review were as follows: (1) the content of the article mainly focused on the provision of brief intervention and/or motivational interview for tobacco use reduction or cessation; (2) the participants were current smokers and adults; (3) the articles were published in peer-reviewed journals within the last ten years; (4) the study method reflected a randomized control trial (RCT). Articles were excluded if they focused primarily on other pharmacologic interventions, included any other substance use, were not designed as an RCT, or had mixed interventions. The search strategy was based on the population, intervention, control, and outcomes (PICO) approach with a PICO question, ‘does motivational interviewing and brief interventions helpful in reducing tobacco use in healthy adults?’; where P- Healthy tobacco users, I- Motivational Interview and/or Brief Intervention, C- Usual care or on other interventions and O- Smoking cessation.(29) A total of 1406 articles were included for a title and abstract review; at least two team members discussed discrepancies. 77 articles met the inclusion criteria for a full-text review, and 12 articles were selected for data extraction. See the PRISMA framework (Figure 1) that guided the review process.(30) 

*Bias assessment.* Cochrane review criteria were used to assess the risk of bias in included studies in the review (Table 1).(31) All studies were evaluated on six evidence-based domains: allocation concealment, random sequence generation, participants and personnel blinding, outcome blinding, incomplete outcome data, and selective reporting.(31) Allocation concealment refers to concealing the information on the randomization process to the subjects. Random sequence generation occurs when study participants are not aware of the random sequence generation process. Blinding of participants and personnel refers to when participants and team members do not know the intervention or control condition to which subjects are assigned. Blinding of outcomes assessment refers to whether outcome measurement could have been changed by prior intervention knowledge to participants or team members delivered in work. Selective reporting refers to presenting only findings of interest. An incomplete outcome does not consider attrition while submitting the result.(31) For each study, these components are shown in ‘high risk,’ ‘low risk,’ or ‘unclear’ as written in the published version of the manuscript to decide on bias assessment. In data extraction, two authors assessed each study for bias. The authors discuss the risk bias criteria of the study using a checklist and conclude. The discrepancies were resolved after a discussion with the third author Table 1. 

**Table 1:** Quality assessment of the included studies

Sources	Random sequence allocation	Allocation concealment	Blinding of participants / personnel	Blinding of outcomes assessment	Complete outcomes data	Avoidance of selective reporting	Quality of study*
Catley *et al*.	✔	✔	X	X	✔	✔	Moderate
Mujika *et al*.	✔	✔	✔	X	✔	✔	High
Virtanen *et al*.	✔	✔	X	X	✔	✔	Moderate
Cook *et al*.	✔	?	✔	X	✔	✔	Moderate
Steinberg *et al.*	✔	?	?	?	✔	✔	Moderate
Ho *et al*.	✔	✔	X	X	✔	✔	Moderate
Cabriale *et al*.	✔	?	?	?	✔	✔	Low
Krigel *et al.*	✔	✔	?	?	✔	✔	Moderate
Meyer *et al.*	✔	?	✔	✔	✔	✔	High
Schane *et al*.	✔	?	?	?	✔	✔	Low
Leavens ELS *et al.*	✔	?	X	?	✔	✔	Low
Cabriales *et al.*	✔	?	X	X	✔	✔	Low

## Results

The electronic search produced a total of 3162 articles. 1406 articles were found suitable after removing duplicate records. Abstracts of all articles were reviewed independently by two reviewers. A total of 1262 articles were excluded after careful scrutiny of abstracts. Full-text articles were retrieved for 79, and after reviewing these articles independently, 67 articles were further excluded for a specific reason. After applying the eligibility criteria, 12 articles were included in the present review. The PRISMA flow diagram (Figure 1) summarizes the study selection and scrutiny process used for the articles. A summary of the selected studies summarized by year of publication, author, setting, type of study, sampling techniques, sample size, eligibility criteria (inclusion and exclusion), intervention, outcomes, strengths and limitations, and any other specific notes to the study. 

Study characteristics. Of the 12 included studies, eight were conducted in the United States, one in Sweden, one in Hong Kong, one in Germany, and one in Spain. All studies used a randomized controlled trials design with one or another trial feature, including allocation concealment and blinding. Of the 12 studies, 3 studies used brief intervention or brief advise,(30,32,35,38,41) 6 studies used motivational interviews (15,30,33,34,37,40), and one study used brief counseling on harm to self and harm to others (39) and quit immediately award model based on brief intervention approach. Seven of the 12 studies (58.3%) reported a beneficial effect of brief advice or motivational interview on reducing tobacco use (Table 2).

Motivational Interviewing (MI). The concept and use of motivational interviewing as an intervention is not new in substance use,(42) smoking reduction,(43) chronic lifestyle disease,(44) health behavior,(45) medication adherence,(46,47) oral health in adolescents,(48) and chronic pain management.(49) The concept was published by Miller & Rollnick and presented as a therapeutic effort to strengthen personal motivation and commitment to a specific goal by eliciting and exploring the individual’s reason for a change in behavior with compassion and acceptance.(16) 

Motivational interviewing (MI) is a patient-centered, directive therapeutic style to improve readiness to change behavior by resolving the ambivalence.(43) MI was found to be an effective method in a series of addictive behaviors.(50) Some research(33) among healthy adult smokers tested multiple interventions revealed a promising effect of motivational interviewing on smoking reduction. However, the study concluded(50) that motivational interviewing and other interventions will produce the most consistent and marked reduction in smoking. A contrasting study(15) used motivational interviewing over health education and brief advice but did not report any change in quit attempts at 6 months. However, the same study reported increased cessation of medication use, motivation, and confidence to quit compared to brief advice, which further indicates the effectiveness of MI in behavior changes to quit smoking. In a study(34) at a Northeastern US State, daily smokers attended brief motivational interviewing and significantly reduced cigarette use. Likewise, motivational interviewing effectively improved quitting smoking among nurses over brief advice in a study conducted in Spain.(30) However, in another work(37) on college tobacco smokers, the use of motivational interviewing over health education (HE) showed no significant reduction in motivation to quit, abstinence, and quit attempts. Likewise, the consistent findings are presented in earlier studies(15,51) that reported no significant advantage of MI on smoking cessation compared to alternative interventions. In a recent work conducted in the Midwest United States, a brief motivational interview showed no improvement in reducing water pipe use(40); however, MI was found to improve awareness of risk perceptions, commitment, and confidence to quit waterpipe (WP) smoking.

Furthermore, in a recent meta-analysis, MI reported a modest yet significant beneficial increase in quitting rates in a group that utilized motivational interviewing. Further, findings revealed that long-term motivational interviewing by a primary physician or counselor is more effective in quitting tobacco. However, there is no specific evidence on the duration and number of MI sessions on quitting the behavior. Another meta-analysis(52) reported a greater likelihood of abstinence behavior in the experimental arm comprising adults and adolescents when compared to the comparison group. Still, only a few older interventions and meta-analyses demonstrate the effectiveness of motivational interviewing in smoking cessation. There is evidence that motivational interviewing is less effective in low-motivation patients.(18,53) However, the conclusive evidence to prove the quality and fidelity of MI implementation remains contentious concerning its effectiveness in smoking reduction. 

Brief Intervention. Brief intervention or advice for harmful substance use has been practiced for many years. (54) It aims to identify the current and potential problems with substance use and motivate people to change high-risk behavior.(55) Brief intervention is a personalized, supportive and non-judgmental approach to treatment.(55) It is also defined as a verbal ‘stop smoking’ message loaded with harmful effects of tobacco use.(56) Brief intervention can be used in various methodologies, including unstructured counseling and feedback to formal structured treatment.(57-59) World Health Organization uses education, simple advice, and brief counseling as alternative types of brief interventions for high-risk individuals with alcohol use disorders.(60) Brief intervention also uses screening and referral services and is therefore called screening, brief intervention, and referral to treatment (SBIRT).(61) Brief therapy can help motivate an individual to change his high-risk behavior at a different stage of behavior change.(62) The stage of change model proposed by Prochaska & DiClemente, helps clinicians tailor a brief intervention to the stage of behavior change and the client's needs.(63) 

Brief interventions for tobacco use disorders aim to enhance motivation for change and provide evidence-based resources to reduce usage or complete cessation of tobacco products. The 5A’s approach (Ask, Advise, Assess, Assist, & Arrange) is an evidence-based approach that helps tobacco users in different settings with motivational strategies in a systematic fashion.(64) In addition, FLAGS-Feedback, Listen, Advice, Goals, Strategies and ‘FRAMES’-Feedback, Responsibility, Advice, Menu of options, Empathy, and Self-efficacy, are other frameworks used to deliver brief interventions.(65)

The brief intervention is effective in many ways, including cost-effectiveness in terms of time and money,(66) increased abstinence rate and days,(35,67) and early days of discharge, and regular follow-ups (68). Similarly, a more intensive planned brief advice (>20 minutes) may augment the effect on quit rate and 6-months abstinence compared to minimal brief advice.(69) Additionally, the use of brief components in AWARD [Ask, Warn, Advice, Refer, Do-It Again) model, and cut down to quit: [CDTQ]), reported a higher quit rate in the former group. (35) Furthermore, brief advice in combination with tailored practice was highly effective on 7-days point prevalence and 7-days and 6-months abstinence rate among adult smokers. (38) Brief counseling also reported a significant reduction in quit rate, abstinence phenomenon, improved motivation, and self-efficacy in a regular follow-up in a group of nondaily smokers.(36,39) Conversely, brief therapy showed no significant changes in abstinence rate among adults who underwent immediate and delayed intervention at the family health clinic U.S.-Mexico border,(41) and hence, the efficacy of brief therapy has been questioned in recent years.(70) 

Further, brief treatment can be helpful for varied kinds of the population, including adolescents, older smokers, smokers with mental illness and co-morbidities, alcohol users, and pregnant women across different racial and ethnic groups.(66,70) However, current or former tobacco smokers who were willing or unwilling to make quit attempts are the most eligible groups to attend the brief intervention.(66)

## Discussion

The use of tobacco has innumerable adverse effects on health. The present review aimed to assess the effectiveness of a brief intervention in reducing tobacco use among adults. The review findings indicate that brief intervention alone or combined with Motivational Interviews or Health Education was effective, supported by previous results.(15,52) In contrast, an earlier systematic review documented that motivational interviewing was modestly successful in promoting smoking cessation compared with usual care or brief advice.(25) Conversely, motivation to quit was higher after Brief Advice than MI.(71) Another recent systematic review conducted with 37 studies reported insufficient evidence to show whether MI helps people stop smoking compared with no intervention, as an addition to other types of behavioral support, or compared with different kinds of behavioral support for smoking cessation.(72)

Modality and intensity of interventions with follow-up and primary outcomes were also determining factors for the effectiveness of the studies. In the current review, the intervention modality varied in face‐to‐face sessions or a combination of face‐to‐face and telephone sessions. Initial sessions were conducted face-to-face, and the follow-up was done over the telephone for most of the study, which is usual with much other previous work.(72) Brief intervention provided through telephone has great significance in the present scenario. Amid the COVID-19 pandemic, when individuals have restricted movement or limited resources available, virtual or phone delivered brief intervention can play a significant role in helping the adults quit smoking or reduce tobacco use. A previous study has documented moderate‐certainty evidence of proactive telephone counseling in increasing the quit rates in smokers who seek help from quitlines.(73)

The included studies had intervention sessions as little as one brief session(37) to four sessions based on Motivational Interviews. (30) Prior literature suggests that multiple sessions might increase the likelihood of quitting over single-session treatment, but positive outcomes were reported in both cases.(25) However, there is no specific evidence on the duration and number of MI sessions on quitting the behavior.(72)The current review found that the included studies had a follow-up of the intervention ranging from 3 months to 12 months. However, face-to-face or telephone counseling follow-up did not show a significant effect of an intervention. However, reduction of smoking behavior or abstinence was not sustained over time. These findings were supported by a previous work where smoking abstinence averaged 10% at 1 month and around 2% at 3, 6, and 12 months.(71) At present, evidence is unclear on the optimal number of follow-up calls.(25,43)

The primary outcomes of the studies were smoking abstinence, reduction in smoking rates, and an increase in motivation to quit. However, outcomes other than cessation may be essential to assess when determining the effects of brief interventions for tobacco use. Hence, different outcomes were self-efficacy, motivation, and changes in depression over the studies. Biological tests to confirm tobacco abstinence provided more reliable findings than self-reported abstinence. 

Intervention programs on Smoking cessation, such as brief advice, motivational interviews, or the 5A approach (Ask, Advise, Assess, Assist, and Arrange), are effective among specific populations or specialized clinical settings.(45,74) Professional support and cessation interventions or medications significantly increase the chance of successfully quitting.(3) A systematic review and meta-synthesis explored smokers' perspectives regarding smoking cessation and reported that lack of motivation to quit was one of the significant issues they felt for tobacco cessation.(75) Nonetheless, these non-pharmacological interventions had shown efficacy similar to the pharmacological intervention(74) with additional benefits of cost-effectiveness, competency of the provider, and accessibility to the treatment center. 

Tobacco-related deaths and disabilities are increasing around the globe because of the continued use of different kinds of tobacco products. Many earlier studies confirmed the beneficial effect of a brief intervention based on motivational principles to reduce tobacco use. Nurses' role is precise in tobacco cessation to endorse the International Council of Nurses statement to integrate tobacco use prevention and cessation as part of their regular nursing practice.(76) This systematic review indicates the potential benefits of brief intervention, which can be a breakthrough for nurses in tobacco reduction around the globe. However, nursing policymakers should incorporate smoking cessation interventions as a part of standard practice for all the patients. Hence, brief intervention or motivational interviews provide promising results in cessation or reduction of tobacco use which needs to be further supported by evidence.

The present review should be appraised under its many limitations and strengths. Among its strengths is that it provides coverage of randomized controlled trials that included brief intervention and motivational interviewing on smoking and other tobacco use among adults. This review included samples of those with clinical and non-clinical samples using tobacco. The major strength of this review lies in the inclusion of RCT studies that give a clear description of participants' characteristics, methodology, and implemented intervention. Secondly, the risk of bias assessment showed that most studies had low to moderate risk. This review highlights several opportunities for future research, such as brief intervention or motivational interview combined with other adjuncts to improve outcomes and further research integration of these interventions with combination therapies of psychotherapeutic and pharmacological interventions. 

In terms of limitations, the heterogenicity of the selected studies did not allow to reach a specific conclusion. Studies included in this review used different brief intervention and motivational interview forms, making it challenging to synthesize the results and suggest a potential use of these interventions in day-to-day practice. Heterogeneity in population also made it challenging to generalize the findings across all people around the globe. Further, studies involved in the review only investigated tobacco cessation among healthy adults may confer unique limitations on the generalizability of results. The authors suggest interpreting and using review findings cautiously due to variations in treatment fidelity and the inclusion of a limited number of studies. 

## Conclusion.

Over time there have been changes in treatment modalities for tobacco cessation. Preference for non-pharmacological intervention over pharmacological has led the researchers to find supportive evidence. The present review highlights the effectiveness of a brief intervention and motivational interviewing in reducing tobacco use among adults. It also demonstrates that the effects are far-reaching. However, it remains inconclusive which intervention is more effective than the other. Future longitudinal studies or RCTs with direct comparison of different interventions may further refine the evidence-based practice on tobacco cessation among adults.

**Table 2:** Characteristics of included studies in the review

Reference 15: Catley D, Goggin K, Harris KJ, Richter KP, Williams K, Patten C, et al. A randomized trial of motivational interviewing: Cessation induction among smokers with low desire to quit. Am. J. Prev. Med. 2016; 50(5):573-83.
Population and sample size: Setting: Midwestern city, Kansas, USA. Sample: Adult smokers. Sample size: 255. Age (Mean, SD): 45.8 [SD = 10.9]). Design: Single site, parallel-group RCT design. Randomization: Computer-generated random assignment, Imbalanced allocation (2:2:1) for three interventions
Inclusion criteria: Adult age 18 years & currently smoking one or more cigarettes per day, able to speak English, have stable reachability, no intention to get pregnant in the next 6 months, not using any medication for smoking cessation, have no cessation plan in the next 7 days and confirm tobacco use on CO≥7 ppm.
Exclusion criteria: N/A
Intervention and comparators: Motivational interview (MI, n=102) Versus Health education (HE, *n* =102) Versus Brief advise (BA, *n* =52)
Primary outcomes: The health education group significantly shows a higher abstinence rate at 6-month follow-up, Motivational interviews and health education groups showed a more significant increase in reduced medication use, motivation, and confidence to quit over the brief advice group, Health advice was relatively found better to improve motivation than motivational interviewing.
Others: Strengths: Biochemical verification of 7-day smoking point prevalence by saliva testing, use of intensity match comparison design to test the exact effect of MI over health education.
Limitations: Self-reported measures to test motivation, desire to quit, quit attempts, and point prevalence, the study was limited to willing to quit smokers, and findings may not be generalizable to unmotivated smokers.
Any other Notes: Follow-up for all three interventions at 3 months and 6 months. Missing data handling using appropriate measures to avoid bias in the study.
Reference 30: Mujika A, Forbes A, Canga N, de Irala J, Serrano I, Gascó P, et al. Motivational interviewing as a smoking cessation strategy with nurses: an exploratory randomised controlled trial. Int. J. Nurs. Stud. 2014; 51(8):1074-82.
Population and sample size: Setting: Clinical Universidad de Navarra (CUN) in Pamplona (Navarra), teaching hospital, North Spain. Sample: Nurses. Sample size: 30. Age (Mean, SD): 40.15[SD = 9.45]). Design: Two groups parallel experimental design. Randomization: Computer generated random allocation method, and seal the opaque envelope for location concealment.
Inclusion criteria: Nurses who smoke and are ready to participate in the study and nurses work in the hospital irrespective of thinking of quit or not.]
Exclusion criteria: N/A.
Intervention and comparators: Motivational interview (*n* =15)/ brief advices (*n* =15)
Primary outcomes: More nurses in the intervention arm had quit smoking with an absolute difference of 33.3% 95% CI (2.6-58.2). Progress in the stage of changes was more significant in nurses who attended a motivational interview.
Others: Strengths: Biochemical verification of urine cotinine level for recent smoking detection and Micro+Smokerlyzer use for expired Carbon Monoxide (CO) detection for enrollment of the subjects. Detection of self-report of abstinence by biochemically urine cotinine measurement. Intention-to-treat analysis to control bias.
Limitations: Use of self-reported measures to report nicotine dependence, desire, and readiness to quit. Very low small size to study the effectiveness of the intervention. No follow-up to measure smoking cessation. No sample size analysis; small sample size.
Any other Notes: Collection of data at baseline, end of the intervention, and 3 months after the intervention to cross-check adherence. High satisfaction with the acceptability and feasibility of the intervention indicates the genuine interest of the participants. Use of one-to-one sessions with each participant.
Reference 32. Virtanen SE, Zeebari Z, Rohyo I, Galanti MR. Evaluation of a brief counseling for tobacco cessation in dental clinics among Swedish smokers and snus users. A cluster randomized controlled trial (the FRITT study). Prev. Med. 2015; 70:26-32.
Population and sample size: Setting: Gavleborg and Sodermanland county, Sweden. Sample: Patients currently using tobacco daily. Sample size: 467. Age (Mean, SD): 45.57 [SD = 14.91]). Design: Randomized Cluster design. Randomization: Setting randomization with a 1:1 computer-generated random number.
Inclusion criteria: Patient’s age 18-75 years, Daily tobacco users since last 1 year, able to converse in the Swedish language.
Exclusion criteria: Patients with acute dental illness, severe psychiatric disease, alcohol problems, or use illicit drugs and are currently involved in other cessation programs.
Intervention and comparators: Brief advice based on 5A’s principles (*n*=225) Versus usual care (n=242).
Primary outcomes: Reduction of tobacco consumption & changes in the expected direction for all outcomes were more frequent in the intervention arm.
Others: Strengths: The study used brief advice as per standard 5 A’s approach. Selection of big sample size to make the findings generalizable to a similar population.
Limitations: Lack of randomization for patients, use of computer randomized random sequence for only clinics used; lack of blindness and self-report data; failure to screen all eligible patients at some clinics.
Any other Notes: Sub-groups analysis to differentiate the impact of the intervention on snus and smoke users; Demonstration of counseling using interactive teaching techniques; Follow-ups visits after 6- months.
Reference 33: Cook JW, Collins LM, Fiore MC, Smith SS, Fraser D, Bolt DM, et al. Comparative effectiveness of motivation phase intervention components for use with smokers unwilling to quit: a factorial screening experiment. Addiction. 2016; 111(1):117-28.
Population and sample size: Setting: Southern Wisconsin, USA. Sample: Adult smokers. Sample size: 517. Age (Mean, SD): 47.0 ([SD = 14.4]). Design: Balanced four-factor randomized factorial design. Randomization: Stratified permuted, computer-generated block randomization (block size 16) based on gender and clinic.
Inclusion criteria: Adult aged ≥18 years; smoked ≥ 5 cigarettes/day for the previous 6months, adult not interested in quitting in the next 30 days but willing to cut down, able to read, write and speak the English language, agreed to complete assessment, planned to remains in the area for next 6 months, not currently using Bupropion and Varenicline, consented to use only study smoking medication during the study if reported current NRT use; nonmedical contraindications to Nicotine Replacement Therapy (NRT) use, women of potential childbearing agree to use birth control pills.
Exclusion criteria: N/A.
Intervention and comparators: Motivational interviewing vs. none x Nicotine patch vs. none, x Nicotine gum vs. none x Behavioral reduction vs. no intervention (*n*=253) or usual care (*n*=264).
Primary outcomes: Smoking reduction was higher in nicotine gum combined with behavioral reduction counseling group and behavioral reduction counseling combined with motivational interviewing.
Others: Strengths: Use factorial design to test multiple interventions compared to usual care and stratified permuted random sampling. Follow-ups at 12- and 26-weeks following study enrollment.
Limitations: Self-reported response for outcomes measures and limited blinding for staff and participants.
Any other Notes: Use of phase base model of smoking intervention, the use of multiple treatment strategies using factorial design will help to test multiple hypotheses at one time.
Reference 34: Steinberg ML, Rosen RL, Versella M V, Borges A, Leyro TM. A Pilot Randomized Clinical Trial of Brief Interventions to Encourage Quit Attempts in Smokers From Socioeconomic Disadvantage. Nicotine Tob. Res. 2020; 22(9):1500-8.
Population and sample size: Setting: Local community soup kitchen, Northeastern US State. Sample: Daily smokers. Sample size: 64. Age (Mean, SD): (M_age_= 47.4 years [SD = 10.7]). Design: Pilot Randomized Clinical Trial. Randomization: Block randomization.
Inclusion criteria: Patient’s age 19-65 years, daily tobacco users, able to read and speak the English language, and Carbon Monoxide (CO) reading greater than 5 ppm.
Exclusion criteria: Patients on U.S FDA approved smoking cessation aids, patients with severe psychiatric disease, alcohol problems, illicit drug use, and are currently involved in other cessation programs, patients on antipsychotics medications, self-reported current medical problems potential concern to nicotine replacement, pending legal issues with the potential to result in incarceration and women should be on effective birth control and could not be nursing or pregnant or planning to become pregnant in the next 2 months.
Intervention and comparators: Brief (e.g., 30 m) Motivational Interviewing (19), Nicotine Replacement Therapy (NRT) (*n*=19), or a Referral-Only intervention (*n*=20).
Primary outcomes: 40% of the sample reported making a serious quit attempt at follow-up, significant self-reported reduction in smoking and more use of NRT and lozenge in NRT group at 6 months’ follow-up.
Others: Strengths: Unique population (socio-economically disadvantaged smokers), follow-up (30 days) the cases to measure self-reported quit rate/attempt and comparison of three interventions simultaneously in one design.
Limitation: Study included a small sample size (*n*=57).
Any other Notes: Follow-up at 1 month, unique population; socio-economically disadvantaged smokers, use of Post hoc analysis to find financial strain as a significant moderator of the effect of the intervention on smoking behavior
Reference 35: Ho KY, Li WHC, Wang MP, Lam KKW, Lam TH, Chan SSC. Comparison of two approaches in achieving smoking abstinence among patients in an outpatient clinic: A Phase 2 randomized controlled trial. Patient Educ. Couns. 2018; 101(5):885-93.
Population and sample size: Setting: Hong Kong -outpatient clinic. Sample: Chinese smokers- medical follow-up. Sample size: 100. Age (Mean, SD): (M_age_= 55.6 years [SD = NA]). Design: A Phase 2 RCT. Randomization: Computer generated
Inclusion criteria: 18- years or older and smoked at least five cigarettes per day.
Exclusion criteria: Unstable medical conditions, poor cognitive function, mental illness, currently participating in other smoking cessation programs or services.
Intervention and comparators: (Quit immediately: [QI]- received a booklet about smoking cessation and brief intervention using the AWARD [ask, Warn, Advice, Refer, Do-It Again) model, and cut down to quit: [CDTQ]), to quit progressively.
Primary outcomes: QI group had a significantly higher self-reported quit rate than those in the CDTQ group at the 6-monthfollow-up (18.0% vs. 4.0%, adjusted OR = 0.190, 95% CI = 0.039-0.929). Not significant at the 12-month follow-up (12.0% vs. 4.0%, adjusted OR = 0.306, 95% CI = 0.059-1.594).
Others: Strengths: 4 follow-ups (1,3,6,12 months) to measures outcomes, use of allocation concealment to blind randomization and intention-to-treat analysis to control bias in the analysis.
Limitations: A pilot approach to select all subjects from the same setting may infuse participant selection bias and only 6 and 12 months follow up with 73 % retention rate.
Any other Notes: 50 years and over half had received education at the lower secondary school level or below CDTQ methods are relatively more complicated than QI methods, which require an understanding of smoking education strategies and close monitoring of the number of cigarettes consumed and reduced.
Reference 36: Cabriales JA, Suro Maldonado B, Cooper T V. Smoking transitions in a sample of Hispanic daily light and intermittent smokers. Addict Behav. 2016; 62:42-6.
Population and sample size: Setting: Health clinic, hospital, or university on the U.S/México border. Sample: Hispanic (DLS/ITS) daily light (DLS;<=10 cigarettes per day) and intermittent (ITS; nondaily) smokers. Sample size: 190, a subset of 390 follow-up samples. Age (Mean, SD): (M_age_= 38.6 years [SD =15.1]) Design: Randomized controlled trial. Randomization: Randomly assigned to either an immediate or delayed intervention group at baseline using an online random number generator
Inclusion criteria: Age of at least 18 years and smoking between one cigarette a month to 10 cigarettes per day (CPD).
Exclusion criteria: N/A
Intervention and comparators: Immediate brief cessation intervention versus delayed intervention (control) group.
Primary outcomes: Smoking categories to control group (DLS/ITS) remains stable, with no significant group difference. DLS group at both points showed higher nicotine dependence levels.8.95% went from daily light smokers (DLS) to quitting, and 5.26% went from intermittent smokers to quitting at 3-month follow-up.
Others: Strengths: Specific population; Hispanic, an underrepresented population in smoking cessation studies, use of multi-component intervention in one study. The first study to discuss light and intermittent smoking to compare efficacy of brief smoking cessation intervention. 3- month follow-up to measure to measures outcomes in both groups.
Limitations: High attrition rate (48%); “contact-information mobility” - challenges to maintain communication with participants; participant work schedules; prioritization of “personal and family safety” over health-related behaviors; “the study was brief and perhaps not intensive enough to cause cessation.” The self-report method at baseline and follow-up for smoking status rather than biochemical process.
Any other Notes: All-Hispanic, predominantly Mexican/Mexican American community sample potentially limits generalizability.
Reference 37: Krigel SW, Grobe JE, Goggin K, Harris KJ, Moreno JL, Catley D. Motivational interviewing and the decisional balance procedure for cessation induction in smokers not intending to quit. Addict Behav. 2017; 64:171-8.
Population and sample size: Setting: Urban University using the psychology department research pool, USA. Sample: University students. Sample size: 82 Age (Mean, SD): (M_age_= 26.9 years [SD =9.6]) Design: Not Specified [Random assignment of the subjects in two groups]. Randomization: Computer-generated random number assignment in a sealed envelope.
Inclusion criteria: Smoking at least one cigarette during the last 7 days, having no intentions to quit in the next 30 days, age at least 18, college enrollment, and reachability via phone & email.
Exclusion criteria: N/A.
Intervention and comparators: Motivational Interviewing using only the decisional balance component (MIDB)/ health education around smoking cessation (HE).
Primary outcomes: Both groups showed significant reductions in smoking rates and increases in motivation to quit, quit attempts, and self-reported abstinence, with no significant group differences.
Others: Strengths: Cost & time efficient interventions, use of intention-to-treat analysis and maximum-likelihood estimation to accommodate missing data.
Limitations: Population of interest is a small/limited group; “college students who were generally light smokers”. The use of a small sample size may hinder generalizability. Outcomes measures were self-reported without control group with no biochemical verification of abstinence.
Any other Notes: Recruitment materials made no mention of quitting smoking, and participants were informed they would receive up to $20 for study completion. Only one session of MIDB or HE was performed per participant. Each session was, on average <20 minutes.
Reference 38: Meyer C, Ulbricht S, Gross B, Kästel L, Wittrien S, Klein G, et al. Adoption, reach and effectiveness of computer-based, practitioner delivered and combined smoking interventions in general medical practices: a three-arm cluster randomized trial. Drug Alcohol Depend. 2012; 121(1-2):124-32.
Population and sample size: Setting: Northern Eastern, Germany. Sample: Adult smoker patients. Sample size: 263. Age (Mean, SD): 41.17 years [SD = 15.2]). Design: Three-arm clustered randomized controlled design. Randomization: Cluster randomization of the medical practices (*n*=151).
Inclusion criteria: Patients aged more than 18 years or older reported any tobacco smoking use in the last 6 months.
Exclusion criteria: Practices registered for another facility apart from general practice.
Intervention and comparators: Brief advice (practice *n*=50; patients *n*=618)/Tailored letter (practice *n*=50; patients *n*=1484) / Combination (practice *n*=51; patients *n*=1113).
Primary outcomes: The seven-day point prevalence was higher in the combination group compared to brief advice or tailored intervention. The rate of 6- month prolonged was higher in the combination group than the brief advice and tailored letters group. 7-days and 6-month prolonged abstinence were statistically significant between the combination group and the other two groups. Tailored letters group shows significantly higher abstinence within past 7-days at 12-month follow-up in contract to combination and brief advice. The number of abstinent patients was significantly higher in a tailored letter or combination group followed by brief advice.
Others: Strengths: Recruiting a large sample size for a three-arm clustered randomized design. Use of advanced imputations to find best results for ‘missing at random’ cases.
Limitations: Self-reported abstinence and lost to follow-up of one-quarter of patients at 12-months.
Any other Notes: 12 months’ follow-ups for all registered patients. Comparison of three interventions in different arms at a time to determine the efficacy of three different interventions.
Reference 39: Schane RE, Prochaska JJ, Glantz SA. Counseling nondaily smokers about secondhand smoke as a cessation message: a pilot randomized trial. Nicotine Tob. Res. 2013; 15(2):334-42.
Population and sample size: Setting: San Francisco Bay Area, U.S. Sample: Nondaily smokers. Sample size: 52 Age (Mean, SD): 32.66 years [SD = 11.11]). Design: A randomized pilot trial. Randomization: Random sequence created by SAG using the random number generator in Minitab 14.
Inclusion criteria: Respondents smoked at least 100 cigarettes in their lifetime, smoked at least once in the past seven days but not every day, age 18 years or older and speak the English language.
Exclusion criteria: Participants had an exhaled carbon monoxide (CO) exceeding 10ppm.
Intervention and comparators: Brief counseling on Harm to Self-group (HTS, *n* =26) provided information on tobacco use and its risk on developing different medical conditions along with chemical ingredients of tobacco by a nurse/Harm to Others (HTO, *n* =26) informed about tobacco use and its risk on friends and family members similar to the HTS group.
Primary outcomes: A significant difference in abstinence between harm to others (HTO) (36.8%) and harm to self (HTS) (9.5%) groups. A significant change in contemplation ladder score between participants who completed follow-ups than who lost to follow-up. Trying to reduce or quit smoking is higher in the HTO group (not significant, p=0.607). Comparable smoking reduction at 3 months follows in both groups. No difference in intervention acceptability in both the groups. Improved motivation and self-efficacy from baseline to 3-month follow-up in both groups.
Others: Strengths: Bio confirmed tobacco abstinence at the 3- month follow-up.
Limitations: The sample size was small for testing efficacy and limited to self-reported smoking cessation at 3-month follow-up.
Any other Notes: 3-month follow-up for smoking cessation. Bio confirmed tobacco abstinence at the 3 months and use of urinary cotinine test to cross-check the abstinence.
Reference 40: Leavens ELS, Meier E, Tackett AP, Miller MB, Tahirkheli NN, Brett EI, et al. The impact of a brief cessation induction intervention for waterpipe tobacco smoking: A pilot randomized clinical trial. Addict Behav. 2018; 78:94-100.
Population and sample size: Setting: Water pipe (WP) lounges in urban and suburban areas in the Midwest U.S. Sample: Water pipe smokers. Sample size: 109. Age (Mean, SD): 21.1 [SD = 5.08]). Design: Pilot randomized control trial. Randomization: Cluster randomization (block of 4).
Inclusion criteria: Participant age ≥18 years.
Exclusion criteria: N/A.
Intervention and comparators: Brief motivational interview (*n*=53) /No intervention (*n*=55).
Primary outcomes: No Significant difference in WP (number of days WP used and number of WP used). Increase awareness on risk perceptions, commitment to quit, and confidence to quit WP smoking.
Others: Strengths: Cluster randomization to avoid bias in sample selection. Carbon monoxide exposure detection by eCO (exhaled carbon monoxide) detector. Multiple outcome measurement.
Limitations: No eCO detection at 3 months’ follow-up.
Any other Notes: Use of eCO detector at baseline, immediately before entering to lounge and post-session gave more reliable findings. Follow-up survey at 3 months of post-session.
Reference 41: Cabriales JA, Cooper T V., Salgado-Garcia F, Naylor N, Gonzalez E. A randomized trial of a brief smoking cessation intervention in a light and intermittent Hispanic sample. Exp. Clin. Psychopharmacol. 2012; 20(5):410-9.
Population and sample size: Setting: StopLite smoking cessation intervention at a family health clinical (primarily) or university on the U.S. Mexico border. Sample: Hispanic smokers. Sample size: 214. Age (Mean, SD): 38.62 years [SD = 15.08]). Design: Pretest-posttest randomized control-group design with replacement of control group with delayed intervention. Randomization: Online random number generator.
Inclusion criteria: Hispanic at least 18 years of age and smoking between one cigarette a month to 10 cigarettes per day.
Exclusion criteria: Non-Hispanic
Intervention and comparators: Carbon Monoxide (CO) feedback, ME, trigger management, and HE (Immediate versus delayed intervention group).
Primary outcomes: No significant differences in abstinence rates between the immediate and delayed intervention conditions. Significant increases in motivation to quit in the immediate intervention compared to the delayed intervention group.
Others: Strengths: 3-month follow-up by telephone, mail, or in person. Participants in a delayed intervention (control group) received the brief intervention after the end of the study.
Limitations: Self-reported nicotine status as outcome measures and limited to the Hispanic population only.
Any other Notes: The brief intervention included self-efficacy, motivational enhancement, trigger management, and health education components. Non-eligible participants were offered QuitLine & Quintet resources.
